# Acetic Acid Mediated Synthesis of Phosphonate-Substituted Titanium Oxo Clusters

**DOI:** 10.1002/ejic.201400051

**Published:** 2014-03-11

**Authors:** Matthias Czakler, Christine Artner, Ulrich Schubert

**Affiliations:** a Institute of Materials Chemistry, Vienna University of Technology, Getreidemarkt 9, 1060 Wien, Austria

**Keywords:** Cluster compounds, Cage compounds, Titanium, P ligands, Hydrolysis

## Abstract

New phosphonate/acetate-substituted titanium oxo/alkoxo clusters were prepared from Ti(O*i*Pr)_4_ and bis(trimethylsilyl) phosphonates in the presence of acetic acid, which served to generate water in situ through ester formation. The process led to clusters with a higher degree of condensation than in previously known phosphonate-substituted titanium oxo clusters. The clusters [Ti_6_O_4_(O*i*Pr)_10_(OAc)_2_(O_3_PR)_2_] (OAc = acetate) were obtained for a large variety of functional and non-functional groups R under a range of reaction conditions. This cluster type, which is also retained in solution, therefore appears to be very robust. Two other clusters, [Ti_5_O(O*i*Pr)_11_(OAc)(O_3_PCH_2_CH_2_CH_2_Br)_3_] and [Ti_5_O_3_(O*i*Pr)_6_(OAc)_4_(O_3_P-xylyl)_2_], were only isolated in special cases.

## Introduction

Metal alkoxides are often substituted by less easily hydrolyzable organic groups to moderate their reactivity in sol-gel processes and to introduce functional or non-functional organic groups for inorganic–organic hybrid materials.^1^ Such substituted metal alkoxide derivatives are obtained by reacting metal alkoxides with a protic compound, such as β-diketonates, amino alcohols, or oximes. Reaction with carboxylic acids is a special case because this normally does not result in carboxylate-substituted metal alkoxides but, instead, in carboxylate-substituted metal oxo clusters. This is due to the fact that carboxylic acids not only provide carboxylate ligands but also act as an in situ water source through esterification with the eliminated alcohol. Such oxo clusters have been used as nanosized building blocks for the construction of inorganic–organic hybrid polymers or as linker units in metal–organic frameworks (MOF).^2^

Whereas carboxylate-substituted oxo/alkoxo clusters of titanium have been particularly well investigated,^3^ only a few phosphonate-substituted derivatives are known.^4^,^5^ The latter are interesting for hybrid materials because of the strong Ti–O–P bonds, especially when phosphonate ligands with functional organic groups are employed.^6^

We have recently shown that titanium oxo clusters can be easily prepared by using bis(trimethylsilyl) esters.^5^ Compared with the corresponding phosphonic acids, the esters have the advantage that they are soluble in organic solvents. Their reaction with alcohol liberates phosphonic acid, which substitutes part of the OR groups of Ti(OR)_4_ in a relatively fast reaction. Oxo groups are generated in situ by esterification of either coordinated or non-coordinated phosphonic acid, as in the case of carboxylic acids. However, because esterification of phosphonic acids appears to be slow, oxo clusters with a low degree of condensation (defined O/Ti ratio of the Ti/O core^7^) (0.25 or 0.29) were obtained, although higher degrees of condensation can be achieved under solvothermal conditions.^8^

In this article we report the results of experiments in which Ti(O*i*Pr)_4_ was treated with mixtures of various bis(trimethylsilyl) esters of phosphonic acids and acetic acid. The fundamental idea was to increase the proportion of in situ generated water by taking advantage of the easier ester formation of acetic acid. We will show that this approach leads to the formation of titanium oxo clusters substituted by both phosphonate and acetate ligands with an increased degree of condensation.

## Results and Discussion

**Figure 1:**
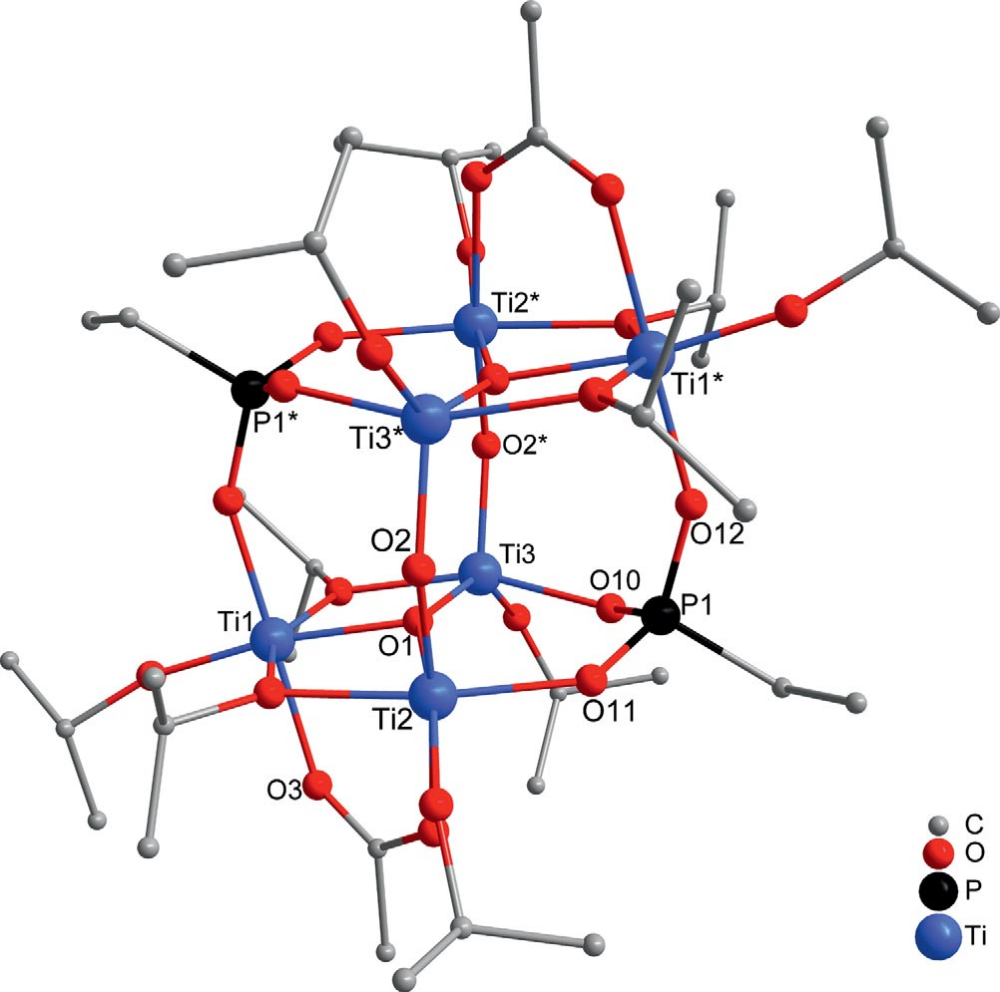
Molecular structure of [Ti_6_(μ_3_-O)_2_(μ_2_-O)_2_(μ_2_-O*i*Pr)_4_(O*i*Pr)_6_(OAc)_2_(O_3_PEt)_2_] (1). Hydrogen atoms are omitted for clarity.

**Figure 2:**
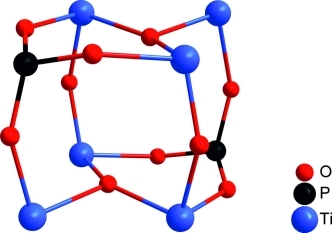
Core structure of the carboxylate-phosphonate-substituted titanium oxo clusters 1–7. Isopropoxo and acetate ligands as well as the substituents at the phosphorus atoms are omitted for clarity.

Reaction of one molar equivalent of bis(trimethylsilyl) ethylphosphonate with two equivalents of acetic acid and four equivalents of Ti(O*i*Pr)_4_ led to the centrosymmetric cluster [Ti_6_(μ_3_-O)_2_(μ_2_-O)_2_(μ_2_-O*i*Pr)_4_(O*i*Pr)_6_(OAc)_2_(O_3_PEt)_2_] (**1**; Figure1, OAc = acetate), with a high degree of condensation (0.67). The cluster was, to the best of our knowledge, also the first mixed carboxylate-phosphonate titanium oxo cluster to be characterized. This new cluster type consists of a Ti_6_O_4_ cluster core with two parallel Ti_3_O triangles connected by μ_2_-oxo and phosphonate bridges. The nearly cubic Ti_6_P_2_O_10_ core resembles that of polyhedral oligomeric silsesquioxanes (POSS) with a Si_8_O_12_ core. The titanium and phosphorus atoms form a distorted parallelepiped (Figure2).

In contrast to the previously obtained clusters with symmetrical, phosphonate-substituted Ti_3_(μ_3_-O)(μ_2_-O*i*Pr)_3_(O*i*Pr)_3_ units as the basic structural motif,^4^,^5^ the structure of **1** is based on unsymmetrically substituted Ti_3_(μ_3_-O)(μ_2_-O*i*Pr)_2_(O*i*Pr)_3_(μ_2_-OAc) units. Two Ti atoms (Ti1 and Ti2) of this unit are bridged by both an O*i*Pr and an acetate ligand, whereas Ti1 and Ti3 are singly bridged by a μ_2_-O*i*Pr group. A terminal O*i*Pr ligand is coordinated to each Ti atom. The two Ti_3_O triangles are connected through a μ_2_-O unit between Ti2 and Ti3* (* denotes the symmetry-related atom in the second Ti_3_O unit) as well as two phosphonate ligands connecting Ti2, Ti3 and Ti1* (and Ti2*, Ti3*, Ti1, respectively). The acetate-bridged atoms Ti1 and Ti2 are thus octahedrally coordinated, whereas Ti3 has a distorted trigonal bipyramidal coordination sphere. Because of the asymmetric substitution of the Ti_3_O triangle, the μ_3_-oxygen (O1) is not in the center of the triangle but has instead a significantly shorter distance to Ti3 [190.61(6) pm] than to the octahedrally coordinated atoms Ti1 and Ti2 [198.04(6) and 199.58(5) pm]. Otherwise, the Ti–O bond lengths at the five-coordinate Ti3 are longer than the corresponding distances of Ti1 and Ti2.

The core of the structure of **1** is comparable to that of [Ti_6_(μ_3_-O)_2_(μ_2_-O)_2_(μ_2_-O*i*Pr)_4_(O*i*Pr)_6_(O_3_SiFlMe)_2_(PhNH_2_)_2_] (Fl = 9-methylfluorenyl),^9^ although all titanium atoms in the titanasiloxane structure are five-coordinate.

In contrast to reactions of Ti(O*i*Pr)_4_ with bis(trimethylsilyl) phosphonates in the absence of acetic acid,^5^ a series of isostructural clusters [Ti_6_O_4_(O*i*Pr)_10_(OAc)_2_(O_3_PR)_2_] was obtained both with much bulkier groups at the phosphorus atom, such as R = CH_2_–naphthyl (**2**), and with functional organic groups, such as R = vinyl (**3**), allyl (**4**), or CH_2_CH_2_CH_2_Cl (**5**) [Equation (1)]. This cluster type therefore appears to be rather robust. With one exception (see below) we obtained this cluster type for the RPO_3_H/HOAc/Ti(O*i*Pr)_4_ ratio of 1:1:2. In some cases this ratio was varied slightly for the preparation of **1**–**5**, from 1:1:2 to 1:2:3 and 1:2:4, but the same cluster was always obtained. Note that, in each case, the molar ratio of (RPO_3_H + HOAc) did not exceed that of Ti(O*i*Pr)_4_.

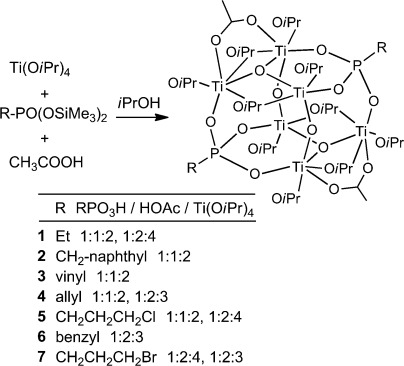
(1)

Clusters **1**–**5** crystallized from the reaction mixture at room temperature within several weeks. To obtain the cluster faster and in higher yield, the synthesis of **1**, as an example, was repeated by heating the reaction mixture (1:2:3) to reflux overnight. The NMR spectra of the resulting powder were the same as those of the sample prepared at room temperature. With this faster preparation process, the isostructural clusters **6** (R = CH_2_Ph) and **7** (R = CH_2_CH_2_CH_2_Br)_2_ were additionally obtained. It can be assumed that clusters **2**–**5** can also be more rapidly obtained by this modification of the synthesis. Cluster **7** was only obtained for RPO_3_H/HOAc/Ti(O*i*Pr)_4_ ratios of 1:2:4 or 1:2:3; surprisingly, for a 1:1:2 ratio another cluster was obtained (see below for cluster **8**).

Clusters **1**–**7** were well-soluble in organic solvents. Their NMR spectra were very similar, and were consistent with the solid-state structures (see the Supporting Information), which shows that the clusters are stable in solution and are not in equilibrium with other structures. The ^1^H NMR spectroscopic data of **1**–**7** show five doublets for the CH_3_ of the O*i*Pr ligands, although the signals of two bridging O*i*Pr ligands overlap at about 1.7 ppm; the other three doublets partly overlap at 1.3–1.5 ppm. For the CH group of the O*i*Pr ligands, three different multiplets were found, in a few cases two of them partly overlap. The singlet for the CH_3_ group of the acetate ligands was observed at about 2.0 ppm. Only one signal between 10 and 30 ppm was observed in the ^31^P NMR spectra, indicating that the clusters are centrosymmetric in solution. ^13^C NMR spectra were in good agreement with the ^1^H NMR spectroscopic data, with six signals for the CH_3_ groups at 23–26 ppm, three signals for the CH groups at 76–79 ppm, and one signal at around 180 ppm for the carboxylate ligand.

**Figure 3:**
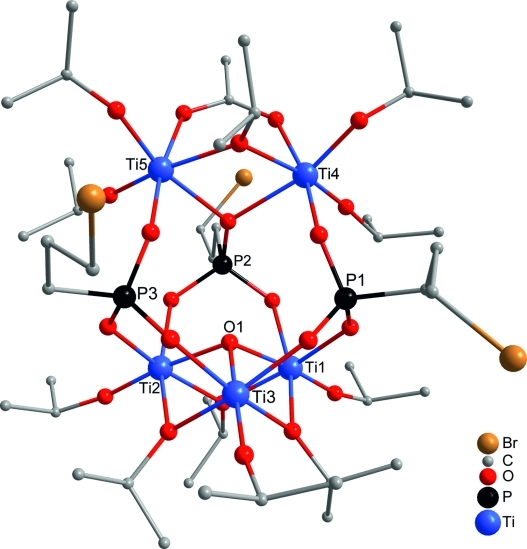
Molecular structure of [Ti_5_(μ_3_-O)(μ_2_-O*i*Pr)_4_(O*i*Pr)_7_(OAc)(O_3_PCH_2_CH_2_CH_2_Br)_3_] (8). Hydrogen atoms are omitted for clarity.

Upon reaction of the aforementioned bis(trimethylsilyl) phosphonates with acetic acid and Ti(O*i*Pr)_4_ in a 1:1:2 ratio, in one case another cluster type was obtained. Reaction of bis(trimethylsilyl) 3-bromopropylphosphonate at room temperature reproducibly resulted in the cluster [Ti_5_(μ_3_-O)(μ_2_-O*i*Pr)_4_(O*i*Pr)_7_(OAc)(O_3_PCH_2_CH_2_CH_2_Br)_3_] (**8**), the structure of which (Figure3) is related to those of previously observed clusters [Ti_4_(μ_3_-O)(μ_2_-O*i*Pr)_3_(O*i*Pr)_5_(O_3_PR)_3_L] (L = neutral ligand).^4^,^5^ The latter consist of a symmetrical Ti_3_(μ_3_-O)(μ_2_-O*i*Pr)_3_(O*i*Pr)_3_ unit to which a Ti(O*i*Pr)_2_L group is connected by means of three phosphonate ligands coordinating to two of the Ti atoms of the Ti_3_O triangle and the capping Ti atom. In **8**, the capping Ti(O*i*Pr)_2_L group is replaced by a Ti_2_(μ_2_-O*i*Pr)(O*i*Pr)_4_(μ_2_-OAc) moiety. Two of the phosphonate ligands are coordinated to only one Ti atom of the Ti_2_ unit, whereas the third bridges both of them. This phosphonate ligand has a binding mode of 4.211 (*w*.*xyz* refers to the number of metal atoms to which the phosphonate ligand is coordinated [*w*], and the number of metal atoms to which each oxygen is coordinated [*x*,*y*,*z*]^10^), whereas the other two phosphonate ligands, as well as those in the cluster **1**–**7**, have a 3.111 binding mode. The Ti_2_(μ_2_-O*i*Pr)(O*i*Pr)_4_(μ_2_-OAc) moiety in **8** is structurally related to Ti_2_(OR)_6_(μ_2_-OOCR′)_2_.^11^

The solution ^1^H NMR spectrum showed several overlapping signals in the region of 1.2–2.0 ppm that can be assigned to the CH_3_ groups of O*i*Pr as well as to the PCH_2_ group. The CH signals of O*i*Pr appear at 4.6–5.4 ppm as five multiplets. The two well-separated triplets for the CH_2_Br group at *δ* = 3.52 and 3.71 ppm have an intensity ratio of 1:2. The same is true for the two resonances at *δ* = 27.44 and 30.34 ppm in the ^31^P NMR spectrum. This is in good agreement with the structure in the crystalline state. Solution ^13^C NMR spectroscopic data confirm the ^1^H NMR spectroscopic data, with corresponding signals at 23–25 ppm for CH_3_ and 77–80 ppm for CH groups. Two doublets were found for each CH_2_ group of the bromopropyl moiety; the signals of the P-CH_2_ groups could not be unequivocally assigned.

**Figure 4:**
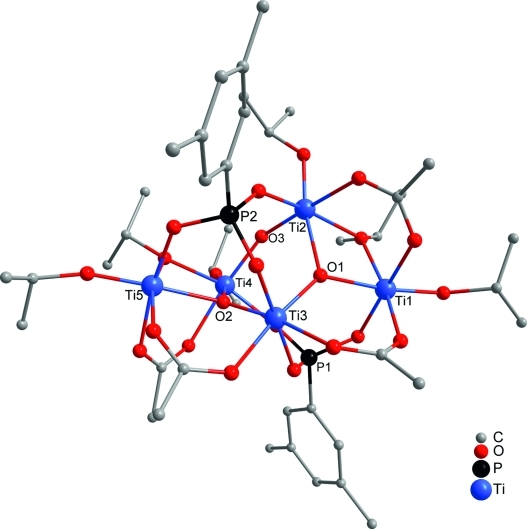
Molecular structure of [Ti_5_(μ_3_-O)_2_(μ_2_-O)(μ_2_-O*i*Pr)_2_(O*i*Pr)_4_(OAc)_4_(O_3_P-xylyl)_2_] (9). Hydrogen atoms are omitted for clarity.

Another Ti_5_ cluster was obtained from the reaction of bis(trimethylsilyl) 3,5-dimethylphenylphosphonate with Ti(O*i*Pr)_4_ and acetic acid (1:2:2). [Ti_5_(μ_3_-O)_2_(μ_2_-O)(μ_2_-O*i*Pr)_2_(O*i*Pr)_4_(OAc)_4_(O_3_P-xylyl)_2_] (**9**; Figure4) consists of five octahedrally coordinated titanium atoms that form two corner-sharing Ti_3_O triangles (Ti1, Ti2, Ti3 and Ti3, Ti4, Ti5), tilted by 52.2°. The cluster has a noncrystallographic *C*_2_ axis passing through the μ_2_-oxygen O3 and Ti5, thus rendering the two Ti_3_O units chemically equivalent. The Ti_3_O units have the composition Ti_3_(μ_3_-O)(μ_2_-O*i*Pr)(O*i*Pr)_2_(μ_2_-OAc)_2_ and can be derived from the basic structural motif Ti_3_(μ_3_-O)(μ_2_-O*i*Pr)_3_(O*i*Pr)_3_ in the previously obtained acetate-free clusters^4^,^5^ and in **8**, and the monosubstituted unit Ti_3_(μ_3_-O)(μ_2_-O*i*Pr)_2_(O*i*Pr)_3_(μ_2_-OAc) in **1**. The Ti_3_O triangles are connected with each other by one μ_2_-O and two phosphonate ligands in a 3.111 binding mode. The central Ti3 atom is coordinated by an oxygen atom of both phosphonate ligands and the other four Ti atoms are coordinated by the oxygen atoms of just one phosphonate ligand.

Cluster **9** has another degree of condensation (0.6) and a higher proportion of acetate ligands than the Ti_5_ cluster **8** or the Ti_6_ clusters **1**–**7**. This is attributed to the higher amount of acetic acid used for the preparation, which apparently led to the higher proportion of acetate ligands in the product. The steric hindrance and the increased acidity of xylylphosphonic acid (aromatic phosphonic acids are slightly more acidic) possibly play an additional role. In previous experiments, when only phosphonates were treated with Ti(O*i*Pr)_4_, the steric bulk of the phosphonate substituents had a significant influence on the cluster structure.^5^

The approximate *C*_2_ symmetry is retained in solution because only one signal was observed in the ^31^P NMR spectrum. The solution ^1^H NMR spectrum show three singlets for the xylyl CH groups at *δ* = 6.91, 8.03 and 8.08 ppm and three multiplets for the isopropoxo CH groups at *δ* = 5.07, 5.22 and 5.66 ppm. One singlet at *δ* = 2.18 ppm was assigned to the CH_3_ groups of the xylyl moieties and two singlets at *δ* = 1.95 and 1.98 ppm to the two acetate ligands. These signals are consistent with respect to shift, number and intensity with the solid-state structure.

Five doublets were found for the CH_3_ groups of the O*i*Pr ligands. Their total intensity corresponded to the calculated value, but only three were expected because of the *C*_2_ symmetry. Two doublets at *δ* = 1.90 and 1.91 ppm overlap with a shift difference of only 0.01 ppm and can be assigned to the two bridging O*i*Pr ligands, which should be symmetry-equivalent. It is therefore assumed that rotation around the O–CH bond is hindered, resulting in different chemical shifts for the two CH_3_ groups. The same can be assumed for one of the terminal CH_3_ groups (possibly interacting with each other) leading to two doublets at *δ* = 1.46 and 1.51 ppm. The other two terminal O*i*Pr ligands show a doublet at *δ* = 1.54 ppm. The same observations were made in the ^13^C NMR spectrum. The increased number of signals in the CH_3_ region is also attributed to sterically hindered rotation of the O*i*Pr groups.

## Conclusions

We have shown that addition of acetic acid to an appropriate mixture of reactants does indeed result in a higher degree of condensation of the obtained oxo clusters compared with the clusters prepared from only bis(trimethylsilyl)phosphonates. This is attributed to the easier esterification of acetic acid compared with phosphonic acids. The higher condensation ratio goes hand in hand with incorporation of acetate ligands in the coordination sphere of the clusters; the new titanium oxo clusters are the first examples of a mixed ligand sphere containing carboxylate, phosphonate and alkoxo ligands.

Whereas reactions of Ti(OR)_4_ with carboxylic acids lead to a great variety of cluster types, depending on the OR group, the acid, and the Ti(OR)_4_/acid ratio,^3^ the reaction with various phosphonates *and* acetic acid led to the same cluster type [Ti_6_O_4_(O*i*Pr)_10_(OAc)_2_(O_3_PR)_2_] (**1**–**7**), which therefore appears to be a rather robust structural entity. The cluster core has an inversion center, and therefore the phosphonate ligands are opposite to each other. Because phosphonate ligands with functional organic groups are easily introduced, reactions of these groups should be possible, e.g., polymerization, thiol-en or addition reactions, by which chains of clusters and hybrid materials with anisotropic structures could be generated.

## Experimental Section

**General Methods:** Manipulations were carried out under an argon atmosphere using standard Schlenk and glove box techniques. Diethyl ethylphosphonate, diethyl (3-bromopropyl)phosphonate, diethyl vinylphosphonate, allyl bromide, 1-bromo-3-chloropropane, benzyl bromide, 1-bromo-3,5-dimethylbenzene, 2-(bromomethyl)naphthalene, triethyl phosphite and Ti(O*i*Pr)_4_ were purchased from Sigma–Aldrich and used as received. Diethyl (3,5-dimethylphenyl)phosphonate was prepared as reported.^12^ Other diethyl phosphonates were prepared by reaction of the corresponding bromide with triethyl phosphite for 16 h in a Dean–Stark apparatus at 150 °C and purified by distillation.

The bis(trimethylsilyl) esters were prepared by adding bromotrimethylsilane (3 equiv.) to a solution of the corresponding diethyl phosphonate (1 equiv.) in CH_2_Cl_2_ followed by removing all volatiles in vacuo and characterization of the compounds by ^31^P and ^1^H NMR spectroscopy.

Isopropyl alcohol was dried by heating to reflux over sodium and distillation. Samples for NMR measurements were obtained by washing the crystalline compounds with isopropyl alcohol and drying. Acetic acid was purified by distillation from P_4_O_10_.

The given yields refer to crystallized compounds. No attempts were made to increase the crystal crop; i.e., the actual yields were higher.

**[Ti_6_O_4_(O*i*Pr)_10_(OAc)_2_(O_3_PEt)_2_] (1):** Bis(trimethylsilyl) ethylphosphonate (184 mg, 0.72 mmol) was added to Ti(O*i*Pr)_4_ (840 μL, 2.9 mmol) in isopropyl alcohol (3 mL). Acetic acid (83 μL, 1.4 mmol) was then quickly added. Cluster **1** crystallized after eight weeks, yield 200 mg (43 %). ^1^H NMR (250 MHz, C_6_D_6_): *δ* = 1.30–1.48 (m, 4 H, CH_2_C*H*_3_), 1.37 (d, *J* = 5.98 Hz, 12 H, CHC*H*_3_), 1.40 (d, *J* = 6.05 Hz, 12 H, CHC*H*_3_), 1.52 (d, *J* = 6.08 Hz, 12 H, CHC*H*_3_), 1.63–1.79 (m, 4 H, CH_2_), 1.78 (d, *J* = 6.28 Hz, 12 H, CHC*H*_3_), 1.84 (d, *J* = 6.30 Hz, 12 H, CHC*H*_3_), 2.00 (s, 6 H, CCH_3_), 4.84 (m, *J* = 6.20 Hz, 2 H, CH), 5.03 (m, *J* = 6.12 Hz, 4 H, CH), 5.36 (m, *J* = 6.28 Hz, 4 H, CH) ppm. ^31^P NMR (101.2 MHz, C_6_D_6_): *δ* = 18.0 ppm. ^13^C NMR (62.9 MHz, C_6_D_6_): *δ* = 7.45 (d, *J* = 6.73 Hz, CH_3_), 20.00 (d, *J* = 158.1 Hz, CH_2_), 23.62 (s, CH_3_), 23.97 (s, CH_3_), 24.25 (s, CH_3_), 24.70 (s, CH_3_), 25.22 (s, CH_3_), 25.31 (s, CH_3_), 77.48 (s, CH), 78.45 (s, CH), 79.21 (s, CH), 177.87 (s, COO) ppm.

**[Ti_6_O_4_(O*i*Pr)_10_(OAc)_2_(O_3_PCH_2_C_10_H_7_)_2_] (2):** Ti(O*i*Pr)_4_ (627 μL, 2.16 mmol) was added to a solution of CH_3_COOH (62 μL, 1.08 mmol) and bis(trimethylsilyl) (naphthylmethyl)phosphonate (396 mg, 1.08 mmol) in isopropyl alcohol (2 mL). After 5 min stirring, the flask allowed to stand for crystallization at room temperature. Crystals of **2** were obtained after three months, yield 200 mg (25 %). ^1^H NMR (400 MHz, C_6_D_6_): *δ* = 1.30 (d, *J* = 5.68 Hz, 12 H, CHC*H*_3_), 1.41 (d, *J* = 6.16 Hz, 12 H, CHC*H*_3_), 1.44 (d, *J* = 5.32 Hz, 12 H, CHC*H*_3_), 1.61 (d, *J* = 6.04 Hz, 12 H, CHC*H*_3_), 1.67 (d, *J* = 5.32 Hz, 12 H, CHC*H*_3_), 1.89 (s, 6 H, CCH_3_), 3.32 (d*, J*_P–H_ = 22.65 Hz, 4 H, PCH_2_), 4.85 (m, *J* = 6.16 Hz, 2 H, CH), 4.95 (m, 4 H, CH), 5.23 (m, *J* = 6.06 Hz, 4 H, CH), 7.33 (t, *J* = 7.43 Hz, 2 H, CH_Ar_), 7.39 (t, *J* = 7.63 Hz, 2 H, CH_Ar_), 7.76 (d*, J* = 8.00 Hz, 2 H, CH_Ar_), 7.84 (d, *J* = 9.04 Hz, 2 H, CH_Ar_), 7.85 (d, *J* = 6.61 Hz, 2 H, CH_Ar_), 7.89 (d, *J* = 7.72 Hz, 4 H, CH_Ar_) ppm. ^31^P NMR (162 MHz, C_6_D_6_): *δ* = 11.58 ppm. ^13^C NMR (100.6 MHz, C_6_D_6_): *δ* = 23.49 (CH*C*H_3_), 23.81 (CH*C*H_3_), 24.16 (CH*C*H_3_), 24.87 (CHC*H*_3_), 25.31 (C*C*H_3_), 35.23 (d, *J* = 151.8 Hz, PCH_2_), 77.75 (*C*HCH_3_), 78.71 (*C*HCH_3_), 79.27 (*C*HCH_3_), 125.10 (CH_Ar_), 125.54 (CH_Ar_), 127.25 (CH_Ar_), 128.74 (d, *J* = 8.78 Hz, CH_Ar_), 129.32 (d, *J* = 4.61 Hz, CCH_2_Ar), 132.48 (CH_Ar_), 132.56 (CH_Ar_), 132.64 (d, *J* = 9.67 Hz, CH_Ar_), 133.87 (C_Ar_), 133.92 (C_Ar_), 177.95 (COO) ppm.

**[Ti_6_O_4_(O*i*Pr)_10_(OAc)_2_(O_3_P-vinyl)_2_] (3):** Bis(trimethylsilyl) vinylphosphonate (0.45 m in CH_2_Cl_2_, 2 mL, 0.9 mmol) was added to Ti(O*i*Pr)_4_ (522 μL, 1.8 mmol) in isopropyl alcohol (1 mL). Acetic acid (51.5 μL, 0.9 mmol) was then quickly added. After five months the CH_2_Cl_2_ was removed, and after an additional two weeks, crystals of **3** were obtained, yield 80 mg (21 %). ^1^H NMR (250 MHz, CDCl_3_): *δ* = 1.38 (d, *J* = 6.16 Hz, 12 H, CHC*H*_3_), 1.39 (d, *J* = 6.16 Hz, 12 H, CHC*H*_3_), 1.52 (d, *J* = 6.10 Hz, 12 H, CHC*H*_3_), 1.80 (d, *J* = 6.32 Hz, 12 H, CHC*H*_3_), 1.84 (d, *J* = 6.32 Hz, 12 H, CHC*H*_3_), 1.99 (s, 6 H, CCH_3_), 4.85 (m, *J* = 6.20 Hz, 2 H, CH), 5.05 (m, *J* = 6.12 Hz, 4 H, CH), 5.38 (m, *J* = 6.28 Hz, 4 H, CH), 5.72 (ddd, *J*_P,H_ = 49.52, *J_trans_* = 12.00, *J_trans_* = 3.48 Hz, 4 H, C*H*=CH_2_), 6.21–6.56 (m, 4 H, CH=C*H*_2_) ppm. ^31^P NMR (101.2 MHz, C_6_D_6_): *δ* = 15.80 ppm. ^13^C NMR (62.9 MHz, C_6_D_6_): *δ* = 23.61 (CH*C*H_3_), 23.97 (CH*C*H_3_), 24.28 (CH*C*H_3_), 24.72 (CH*C*H_3_), 25.26 (CH*C*H_3_), 25.32 (C*C*H_3_), 77.78 (*C*HCH_3_), 78.66 (*C*HCH_3_), 79.49 (*C*HCH_3_), 128.95 (s, CH_2_), 130.69 (d, *J* = 203.96 Hz, *C*H=CH_2_), 177.98 (COO) ppm.

**[Ti_6_O_4_(O*i*Pr)_10_(OAc)_2_(O_3_P-allyl)_2_] (4):** Bis(trimethylsilyl) allylphosphonate (400 mg, 1.6 mmol) was added to Ti(O*i*Pr)_4_ (930 μL, 3.2 mmol) in isopropyl alcohol (2 mL), then acetic acid (91 μL, 1.6 mmol) was quickly added. Crystals of **4** were obtained after three weeks, yield 500 mg (48 %). ^1^H NMR (250 MHz, CD_2_Cl_2_): *δ* = 1.11 (d, *J* = 6.13 Hz, 12 H, CHCH_3_), 1.20 (d, *J* = 6.10 Hz, 12 H, CHCH_3_), 1.34 (d, *J* = 6.23 Hz, 12 H, CHCH_3_), 1.43 (d, *J* = 6.33 Hz, 12 H, CHCH_3_), 1.47 (d, *J* = 6.48 Hz, 12 H, CHCH_3_), 1.90 (s, 6 H, CCH_3_), 2.43 (dd, *J*_H,H_ = 7.4, J_P,H_ = 23.0 Hz, 4 H, CH=CH_2_), 4.73 (m, 6 H, CH), 4.97 (m, 4 H, CH), 5.09 (m, 4 H, CH=CH_2_), 5.90 (m, 2 H, CH=CH_2_) ppm. ^31^P NMR (101.2 MHz, CD_2_Cl_2_): *δ* = 11.9 ppm. ^13^C NMR (62.9 MHz, CD_2_Cl_2_): *δ* = 23.38 (s, CH_3_), 23.61 (s, CH_3_), 23.76 (s, CH_3_), 24.38 (s, CH_3_), 24.77 (s, CH_3_), 24.89 (s, CH_3_), 32.85 (d, *J* = 154.13 Hz, PCH_2_), 77.21 (s, CH), 78.52 (s, CH), 80.05 (s, CH), 117.12 (d, *J* = 14.96 Hz, CH_2_), 130.66 (d, *J* = 11.47 Hz, CH), 177.70 (s, COO) ppm.

**[Ti_6_O_4_(O*i*Pr)_10_(OAc)_2_(O_3_PCH_2_CH_2_CH_2_Cl)_2_] (5):** Bis(trimethylsilyl) (3-chloropropyl)phosphonate (224 mg, 0.74 mmol) was added to Ti(O*i*Pr)_4_ (860 μL, 3.0 mmol) in isopropyl alcohol (3 mL), then acetic acid (85 μL, 1.5 mmol) was quickly added. Cluster **5** crystallized after three weeks, yield 100 mg (20 %). ^1^H NMR (250 MHz, CD_2_Cl_2_): *δ* = 1.13 (d, *J* = 6.00 Hz, 12 H, CHC*H*_3_), 1.21 (d, *J* = 5.95 Hz, 12 H, CHC*H*_3_), 1.35 (d, *J* = 6.05 Hz, 12 H, CHC*H*_3_), 1.45 (d, *J* = 5.80 Hz, 12 H, CHC*H*_3_), 1.47 (d, *J* = 5.33 Hz, 12 H, CHC*H*_3_), 1.71 (m, *J*_H,H_ = 7.30, *J*_P,H_ = 18.72 Hz, 4 H, PCH_2_), 1.92 (s, 6 H, CCH_3_), 2.15 (m, 4 H, CH_2_), 3.75 (t, *J* = 6.71 Hz, 4 H, CH_2_Cl), 4.76 (m, *J* = 6.58 Hz, 6 H, CH), 4.98 (m, *J* = 6.16 Hz, 4 H, CH) ppm. ^31^P NMR (101.2 MHz, CD_2_Cl_2_): *δ* = 15.44 ppm. ^13^C NMR (62.9 MHz, CD_2_Cl_2_): *δ* = 23.59 (s, CH_3_), 23.91 (s, CH_3_), 24.25 (s, CH_3_), 24.37 (d, *J* = 157.3 Hz, PCH_2_), 24.71 (s, CH_3_), 25.28 (s, CH_3_), 27.57 (d, *J* = 4.94 Hz, CH_2_), 44.94 (d, *J* = 12.97 Hz, CH_2_Cl), 77.82 (s, CH), 78.70 (s, CH), 79.54 (s, CH), 178.13 (s, COO) ppm.

**[Ti_6_O_4_(O*i*Pr)_10_(OAc)_2_(O_3_PCH_2_Ph)_2_] (6):** Ti(O*i*Pr)_4_ (2.8 mL, 9.5 mmol) and acetic acid (365 μL, 6.4 mmol) were added to a solution of bis(trimethylsilyl) benzylphosphonate (1 g, 3.2 mmol) in isopropyl alcohol (20 mL). After heating to reflux overnight and cooling to room temperature, a cloudy mixture was obtained. The suspension was concentrated under vacuum and filtered. After washing two times with small portions of *i*PrOH and drying, a white powder of **6** was obtained. Part of the powder was crystallized from CH_2_Cl_2_, yield 1 g (45 %). ^1^H NMR (250 MHz, CDCl_3_): *δ* = 1.34 (d, *J* = 6.13 Hz, 12 H, CHC*H*_3_), 1.42 (d, *J* = 6.20 Hz, 12 H, CHC*H*_3_), 1.47 (d, *J* = 6.10 Hz, 12 H, CHC*H*_3_), 1.67 (d, *J* = 6.25 Hz, 12 H, CHC*H*_3_), 1.73 (d, *J* = 6.23 Hz, 12 H, CHC*H*_3_), 1.98 (s, 6 H, CCH_3_), 3.19 (d*, J*_P,H_ = 22.54 Hz, 4 H, PCH_2_), 4.87 (m, *J* = 6.21 Hz, 2 H, CH), 4.97 (m, *J* = 6.04 Hz, 4 H, CH), 5.25 (m, *J* = 6.24 Hz, 4 H, CH), 7.2 (m, 2 H, CH_Ph_), 7.33 (t, *J* = 7.66 Hz, 4 H, CH_Ph_), 7.64 (d, *J* = 7.49 Hz, 4 H, CH_Ph_) ppm. ^31^P NMR (101.2 MHz, CDCl_3_): *δ* = 23.6 ppm. ^13^C NMR (62.9 MHz, C_6_D_6_): *δ* = 23.62 (CH*C*H_3_), 23.84 (CH*C*H_3_), 24.20 (CH*C*H_3_), 24.86 (CH*C*H_3_), 25.35 (C*C*H_3_), 34.94 (d, *J* = 152.2 Hz, PCH_2_), 77.73 (*C*HCH_3_), 78.69 (*C*HCH_3_), 79.16 (*C*HCH_3_), 125.83 (d, *J* = 2.96 Hz, CH_Ph_), 130.51 (d, *J* = 6.82 Hz, CH_Ph_), 134.91 (d, *J* = 9.07 Hz, C_Ar_CH_2_), 177.88 (COO) ppm.

**[Ti_6_O_4_(O*i*Pr)_10_(OAc)_2_(O_3_PCH_2_CH_2_CH_2_Br)_2_] (7):** Ti(O*i*Pr)_4_ (29 mL, 100 mmol) and acetic acid (3.83 mL, 67 mmol) were added to a solution of bis(trimethylsilyl) (3-bromopropyl)phosphonate (11.63 g, 33.4 mmol) in *i*PrOH (50 mL). After heating to reflux overnight and cooling to room temperature, a cloudy mixture was obtained. The suspension was concentrated under vacuum and filtered. After washing two times with *n*-hexane and drying, a white powder of **7** was obtained. For single-crystal measurements, part of the powder was crystallized from CH_2_Cl_2_, yield 8 g (33 %). ^1^H NMR (250 MHz, C_6_D_6_): *δ* = 1.37 (d, *J* = 6.15 Hz, 12 H, CHC*H*_3_), 1.41 (d, *J* = 6.40 Hz, 12 H, CHC*H*_3_), 1.51 (d, *J* = 6.08 Hz, 12 H, CHC*H*_3_), 1.71 (m, 4 H, PCH_2_), 1.76 (d, *J* = 6.16 Hz, 12 H, CHC*H*_3_), 1.82 (d, *J* = 5.90 Hz, 12 H, CHC*H*_3_), 2.04 (s, 6 H, CCH_3_), 2.35 (m, *J*_H,H_ = 7.23, *J*_P,H_ = 17.30 Hz, 4 H, CH_2_), 3.52 (t, *J* = 7.35 Hz, 4 H, CH_2_Br), 4.85 (m, *J* = 6.20 Hz, 2 H, CH), 5.00 (m, *J* = 6.00 Hz, 4 H, CH), 5.33 (m, *J* = 6.28 Hz, 4 H, CH) ppm. ^31^P NMR (101.2 MHz, C_6_D_6_): *δ* = 15.5 ppm. ^13^C NMR (62.9 MHz, C_6_D_6_): *δ* = 23.64 (s, CH_3_), 23.91 (s, CH_3_), 24.25 (s, CH_3_), 24.74 (s, CH_3_), 25.29 (s, CH_3_), 25.33 (s, CH_3_), 25.71 (d, *J* = 157.2 Hz, PCH_2_), 27.85 (d, *J* = 4.49 Hz, CH_2_), 33.71 (d, *J* = 12.98 Hz, CH_2_Br), 77.83 (s, CH), 78.70 (s, CH), 79.54 (s, CH), 178.14 (s, COO) ppm.

**[Ti_5_O(O*i*Pr)_11_(OAc)(O_3_PCH_2_CH_2_CH_2_Br)_3_] (8):** Bis(trimethylsilyl) (3-bromopropyl)phosphonate (233 mg, 0.67 mmol) was added to Ti(O*i*Pr)_4_ (388 μL, 1.34 mmol) in isopropyl alcohol (2 mL), then acetic acid (38.3 μL, 0.67 mmol) was quickly added. Cluster **8** crystallized after 12 weeks, yield 100 mg (26 %). ^1^H NMR (250 MHz, C_6_D_6_): *δ* = 1.38 (d, *J* = 7.90 Hz, 6 H, CHC*H*_3_), 1.41 (d, *J* = 6.40 Hz, 6 H, CHC*H*_3_), 1.45 (d, *J* = 6.15 Hz, 18 H, CHC*H*_3_), 1.51 (d, *J* = 6.05 Hz, 6 H, CHC*H*_3_), 1.63 (d, *J* = 6.30 Hz, 12 H, CHC*H*_3_), 1.79 (m, 18 H, CHC*H*_3_), 1.85–2.01 (m, 6 H, PCH_2_), 2.04 (s, 3 H, CCH_3_), 2.29–2.54 (m, 6 H, CH_2_), 3.52 (t, *J* = 7.23 Hz, 2 H, CH_2_Br), 3.71 (t, *J* = 6.40 Hz, 4 H, CH_2_Br), 4.69 (m, *J* = 6.20 Hz, 2 H, CH), 4.84 (m, *J* = 6.28 Hz, 1 H, CH), 5.00 (m, *J* = 6.16 Hz, 2 H, CH), 5.16 (m, *J* = 6.44 Hz, 3 H, CH), 5.33 (m, *J* = 6.24 Hz, 3 H, CH) ppm. ^31^P NMR (101.2 MHz, C_6_D_6_): *δ* = 27.44 (1 P), 30.34 (2 P) ppm. ^13^C NMR (62.9 MHz, C_6_D_6_): *δ* = 23.65 (s, CH_3_), 23.96 (s, CH_3_), 24.26 (s, CH_3_), 24.49 (s, CH_3_), 24.74 (s, CH_3_), 24.81 (s, CH_3_), 25.22 (s, CH_3_), 25.31 (s, CH_3_), 26.95 (d, *J* = 4.52 Hz, CH_2_), 27.86 (d, *J* = 4.99 Hz, CH_2_), 33.73 (d, *J* = 12.59 Hz, CH_2_Br), 35.82 (d, *J* = 15.06 Hz, CH_2_Br), 77.84 (s, CH), 78.49 (s, CH), 78.73 (s, CH), 79.57 (s, CH), 79.82 (s, CH), 178.17 (s, COO) ppm.

**[Ti_5_O_3_(O*i*Pr)_6_(OAc)_4_(O_3_P-xylyl)_2_] (9):** Bis(trimethylsilyl) xylylphosphonate (100 mg, 0.30 mmol) was added to a solution of Ti(O*i*Pr)_4_ (176 μL, 0.61 mmol) in isopropyl alcohol (1 mL), then CH_3_COOH (35 μL, 0.61 mmol) was added. After 15 weeks, crystals of **9** were obtained, yield 200 mg (48 %). ^1^H NMR (250 MHz, C_6_D_6_): *δ* = 1.46 (d, *J* = 6.15 Hz, 6 H, CHC*H*_3_), 1.51 (d, *J* = 6.78 Hz, 6 H, CHC*H*_3_), 1.54 (d, *J* = 6.33 Hz, 12 H, CHC*H*_3_), 1.90 (d, *J* = 6.20 Hz, 6 H, CHC*H*_3_), 1.91 (d, *J* = 6.32 Hz, 6 H, CHC*H*_3_), 1.95 (s, 6 H, CCH_3_), 1.98 (s, 6 H, CCH_3_), 2.18 (s, 12 H, CH_3(Xyl)_), 5.07 (m, *J* = 6.20 Hz, 2 H, CH), 5.22 (m, *J* = 6.12 Hz, 2 H, CH), 5.66 (m, *J* = 6.32 Hz, 2 H, CH), 6.91 (s, 2 H, CH_Xyl_), 8.03 (s, 2 H, CH_Xyl_), 8.08 (s, 2 H, CH_Xyl_) ppm. ^31^P NMR (101.2 MHz, C_6_D_6_): *δ* = 15.92 ppm. ^13^C NMR (62.9 MHz, C_6_D_6_): *δ* = 20.92 (CH_3_), 22.90 (CH_3_), 23.30 (CH_3_), 23.93 (CH_3_), 24.05 (CH_3_), 24.11 (CH_3_), 24.16 (CH_3_), 24.60 (CH_3_), 24.77 (CH_3_), 25.19 (CH_3_), 80.57 (*C*HCH_3_), 81.41 (*C*HCH_3_), 82.27 (*C*HCH_3_), 129.80 (d, *J* = 10.0 Hz, CH), 131.44 (d, *J* = 201.0 Hz, PC), 132.53 (d, *J* = 2.8 Hz, CH), 137.24 (d, *J* = 16.3 Hz, C), 177.87 (COO), 180.11 (COO) ppm.

**X-ray Structure Analyses:** All measurements were performed at 100 K using Mo-*K*_α_ (*λ* = 71.073 pm) radiation. Data were collected with a Bruker AXS SMART APEX II four-circle diffractometer with κ-geometry. Crystals of **5** cracked when cooled to 100 K; apparently a phase transition took place at about 180 K. The measurements of **5** were therefore carried out at 213 K. Data were collected with *φ* and *ω*-scans and different frame widths. The data were corrected for polarization and Lorentz effects, and an empirical absorption correction (SADABS) was employed. The cell dimensions were refined with all unique reflections. SAINT PLUS software (Bruker Analytical X-ray Instruments, 2007) was used to integrate the frames. Details of the X-ray investigations are given in Table1 (for **1**–**3**), Table2 (for **4**–**6**), and Table3 (for **7**–**9**). The structures were solved by the Patterson method (SHELXS97^13^). Refinement was performed by the full-matrix least-squares method based on *F*^2^ (SHELXL97) with anisotropic thermal parameters for all non-hydrogen atoms. Hydrogen atoms were inserted in calculated positions and refined by riding with the corresponding atom. Positions of disordered carbon and halogen atoms were refined with two sites, with about 50 % occupancy each. Disordered groups: three O*i*Pr ligands of **1**; one vinyl group of **3**; three O*i*Pr ligands and one chloropropyl group of **5**; four O*i*Pr ligands and one dichloromethane of **6**; four O*i*Pr ligands of **9**.

**Table 1:** Crystal data and structure refinement details for 1–9.

	1	2	3
Empirical formula	C_38_H_86_O_24_P_2_Ti_6_	C_56_H_94_O_24_P_2_Ti_6_	C_38_H_82_O_24_P_2_Ti_6_
Formula weight	1276.22	1500.65	1272.38
Crystal system	monoclinic	monoclinic	monoclinic
Space group	*P* 2_1_/*n*	*C* 2/*c*	*P* 2_1_/*n*
*a* [pm]	1226.23(6)	2259.5(2)	1206.87(6)
*b* [pm]	1649.58(9)	1682.61(13)	1664.31(8)
*c* [pm]	1464.69(7)	1830.97(15)	1460.95(8)
*α* [°]	90	90	90
*β* [°]	98.272(3)	91.538(3)	97.193(2)
*γ* [°]	90	90	90
*V* (pm^3^**·**10^6^)	2931.9(3)	6958.5(10)	2911.4(3)
Z	2	4	2
*D_x_* [Mg m^–3^]	1.446	1.432	1.451
μ [mm^–1^]	0.904	0.774	0.91
Crystal size [mm]	0.5 × 0.5 × 0.5	0.5 × 0.5 × 0.4	0.5 × 0.4 × 0.3
Measured reflections	121208	93315	43475
Observed reflections [*I* > 2σ(*I*)]	16514	10942	5034
*θ*_max._ [°]	42.84	33.22	26.41
*R* [*F*^2^ > 2σ(*F*)], *wR*(*F*^2^), *S*	0.0323, 0.0862, 1.037	0.0356, 0.1004, 1.043	0.0424, 0.1002, 1.106
Reflections/parameters	21197/418	13284/408	5971/448
Weighting scheme[a]	*w* = 1/[σ^2^(*F*_o_^2^) + (0.0344*P*)^2^ + 1.21*P*]	*w* = 1/[σ^2^(*F*_o_^2^) + (0.0417*P*)^2^ + 14.8148*P*]	*w* = 1/[σ^2^(*F*_o_^2^) + (0.0344*P*)^2^ + 9.7385*P*]
*δρ*_max.,min._ [e**·**10^–6^ pm^–3^]	0.885, –1.307	1.246, –0.424	1.641, –0.957

[a] *P* = (*F*_o_^2^ + 2*F*_c_^2^)/3.

**Table 2:** Crystal data and structure refinement details for 4–6.

	4	5	6
Empirical formula	C_40_H_86_O_24_P_2_Ti_6_	C_40_H_88_Cl_2_O_24_P_2_Ti_6_	C_50_H_94_Cl_4_O_24_P_2_Ti_6_
Formula weight	1300.43	1373.34	1570.39
Crystal system	triclinic	monoclinic	monoclinic
Space group	*P* 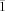	*P* 2_1_/n	*P*2_1_/*c*
*a* [pm]	1146.80(9)	1376.54(5)	2643.8(3)
*b* [pm]	1223.83(10)	1571.04(5)	1259.66(14)
*c* [pm]	1228.22(10)	1653.85(5)	2280.3(3)
*α* [°]	106.880(4)	90	90
*β* [°]	110.202(3)	112.1300(10)	108.761(4)
*γ* [°]	97.601(4)	90	90
*V* (pm^3^**·**10^6^)	1495.2(2)	3313.13(19)	7190.8(14)
Z	1	2	4
*D_x_* [Mg m^–3^]	1.444	1.377	1.451
μ [mm^–1^]	0.888	0.883	0.896
Crystal size [mm]	0.3 × 0.3 × 0.3	0.5 × 0.4 × 0.4	0.32 × 0.27 × 0.23
Measured reflections	62482	88173	287456
Observed reflections [*I* > 2σ(*I*)]	12261	5416	16780
*θ*_max._ [°]	36.35	28.95	30.58
*R* [*F*^2^ > 2σ(*F*)], *wR*(*F*^2^), *S*	0.0263, 0.0726, 1.036	0.0647, 0.2177, 1.094	0.044, 0.1256, 1.068
Reflections/parameters	14474/336	8720/443	21997/894
Weighting scheme[a]	*w* = 1/[σ^2^(*F*_o_^2^) + (0.0313*P*)^2^ + 0.6556*P*]	*w* = 1/[σ^2^(*F*_o_^2^) + (0.0889*P*)^2^ + 3.5722*P*]	*w* = 1/[σ^2^(*F*_o_^2^) + (0.0517*P*)^2^ + 11.3637*P*]
*δρ*_max.,min._ [e**·**10^–6^ pm^–3^]	1.272, –0.362	0.741, –0.477	1.5, –1.368

[a] *P* = (*F*_o_^2^ + 2*F*_c_^2^)/3.

**Table 3:** Crystal data and structure refinement details for 7–9.

	7	8	9
Empirical formula	C_40_H_88_Br_2_O_24_P_2_Ti_6_	C_44_H_98_Br_3_O_23_P_3_Ti_5_	C_42_H_72_O_23_P_2_Ti_5_
Formula weight	1462.26	1567.36	1246.44
Crystal system	orthorhombic	monoclinic	triclinic
Space group	*P b c a*	*P* 2_1_/*n*	*P* 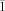
*a* [pm]	1632.40(3)	1308.99(7)	1210.96(6)
*b* [pm]	1585.18(3)	2291.37(13)	1284.46(7)
*c* [pm]	2401.10(4)	2346.81(13)	1908.81(11)
*α* [°]	90	90	101.464(3)
*β* [°]	90	102.299(2)	96.661(3)
*γ* [°]	90	90	96.627(3)
*V* (pm^3^**·**10^6^)	6213.2(2)	6877.4(7)	2860.8(3)
Z	4	4	2
*D_x_* [Mg m^–3^]	1.563	1.514	1.447
μ [mm^–1^]	2.142	2.434	0.797
Crystal size [mm]	0.6 × 0.5 × 0.5	0.6 × 0.4 × 0.35	0.7 × 0.6 × 0.4
Measured reflections	196724	127718	137433
Observed reflections [*I* > 2σ(*I*)]	10744	13683	19253
*θ*_max._ [°]	34.37	28.32	36.37
*R* [*F*^2^ > 2σ(*F*)], *wR*(*F*^2^), *S*	0.0222, 0.0571, 1.049	0.0567, 0.1487, 1.031	0.063, 0.1544, 1.084
Reflections/parameters	13047/345	16623/727	27635/789
Weighting scheme[a]	*w* = 1/[σ^2^(*F*_o_^2^) + (0.0251*P*)^2^ + 2.6246*P*]	*w* = 1/[σ^2^(*F*_o_^2^) + (0.0552*P*)^2^ + 46.4092*P*]	*w* = 1/[σ^2^(*F*_o_^2^) + (0.0160*P*)^2^ + 9.9669*P*]
*δρ*_max.,min._ [e**·**10^–6^ pm^–3^]	0.681, –0.423	5.461, –5.769	1.701, –1.112

[a] *P* = (*F*_o_^2^ + 2*F*_c_^2^)/3.

CCDC-981140 (for **1**), -CCDC-981141 (for **2**), -CCDC-981142 (for **3**), -CCDC-981143 (for **4**), -CCDC-981144 (for **5**), -CCDC-981145 (for **6**), -CCDC-981146 (for **7**), -CCDC-981147 (for **8**), and -CCDC-981148 (for **9**) contain the supplementary crystallographic data for this paper. These data can be obtained free of charge from The Cambridge Crystallographic Data Center via www.ccdc.cam.ac.uk/data_request/cif.

## Supporting Information

**Supporting Information** (see footnote on the first page of this article): Copies of ^1^H and ^13^C NMR spectra.

miscellaneous_information
