# Supplementary material for: Acetic Acid Mediated Synthesis of Phosphonate-Substituted Titanium Oxo Clusters
Source: Eur J Inorg Chem. 2014 Mar 11;2014(12):2038–45. doi: 10.1002/ejic.201400051 (PMC4362471; doi:10.1002/ejic.201400051)

Eur. J. Inorg. Chem. **2014** · © WILEY-VCH Verlag GmbH & Co. KGaA, 69451 Weinheim, 2014 · ISSN 1099–0682

SUPPORTING INFORMATION

DOI: 10.1002/ejic.201400051
Title: Acetic Acid Mediated Synthesis of Phosphonate-Substituted Titanium Oxo Clusters
Author(s): Matthias Czakler, Christine Artner, Ulrich Schubert*

^{1}H NMR spectrum of **1**

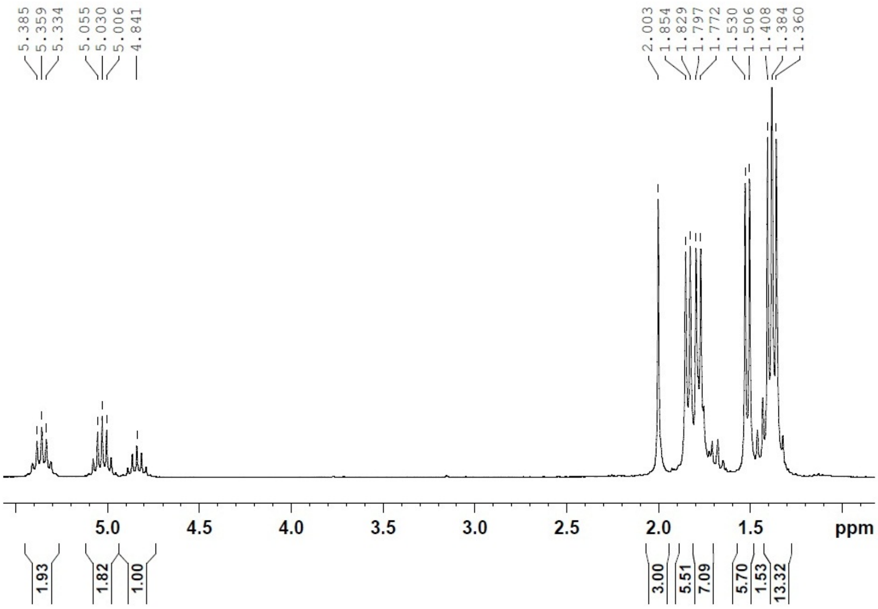

^{13}C NMR spectrum of **1**

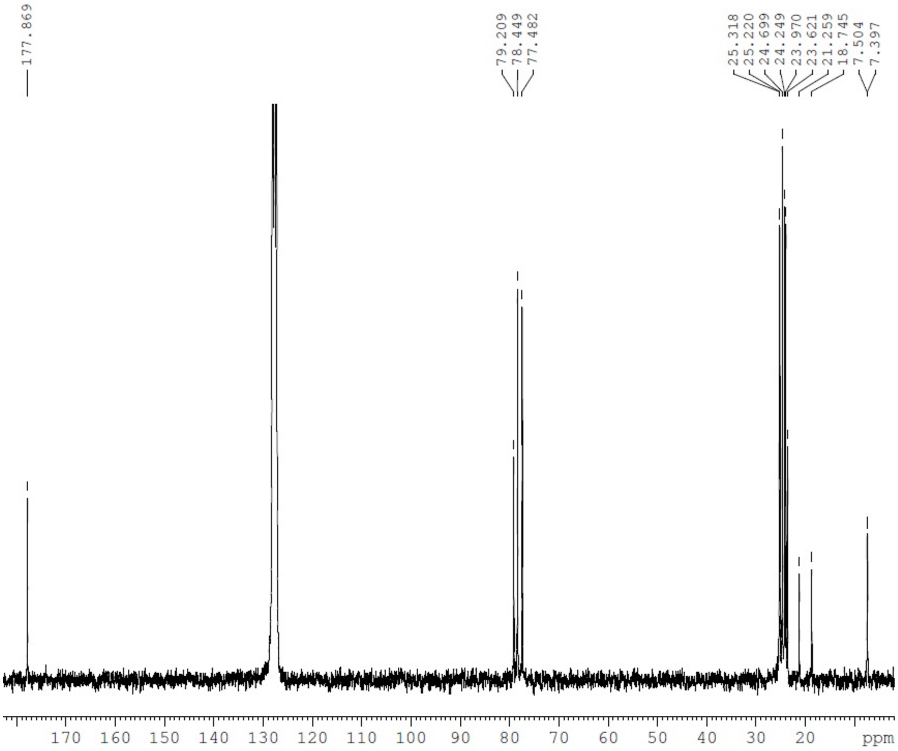

^{1}H NMR spectrum of **4**

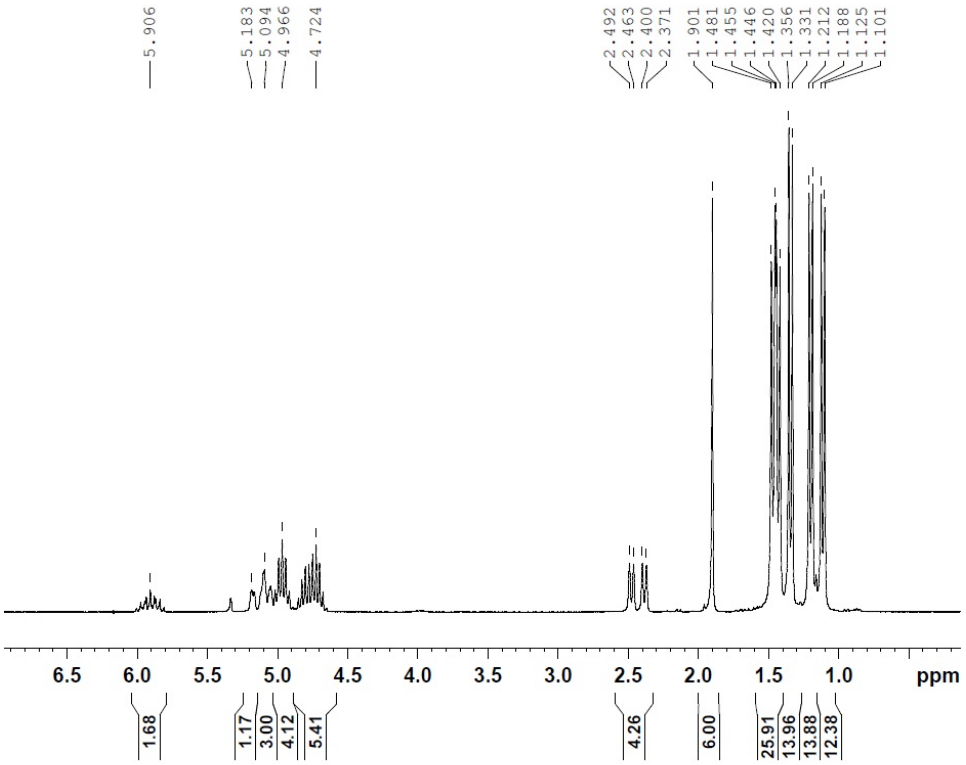

^{13}C NMR spectrum of **4**

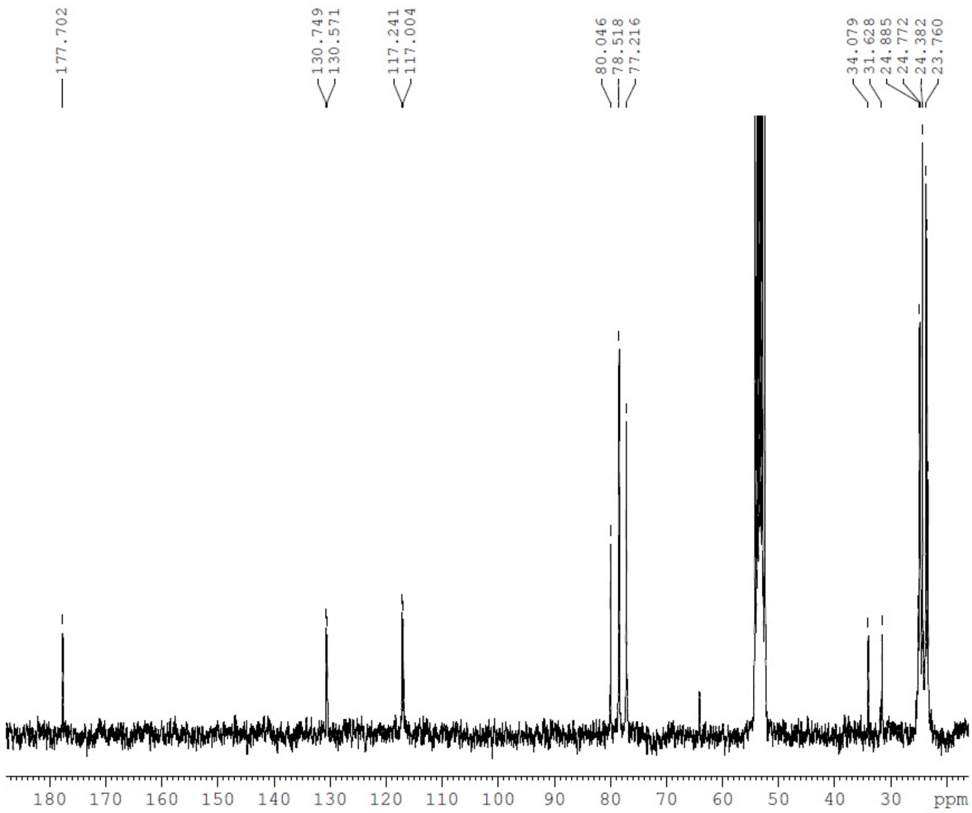

^{1}H NMR spectrum of **7**

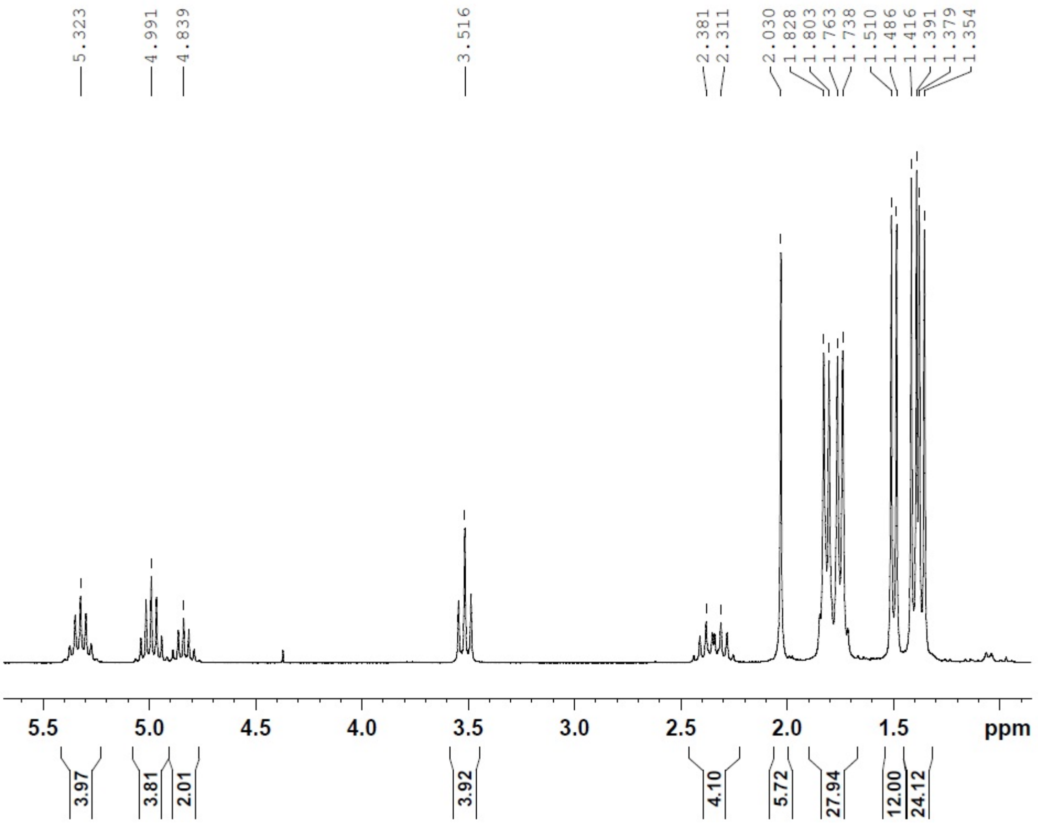

^{13}C NMR spectrum of **7**

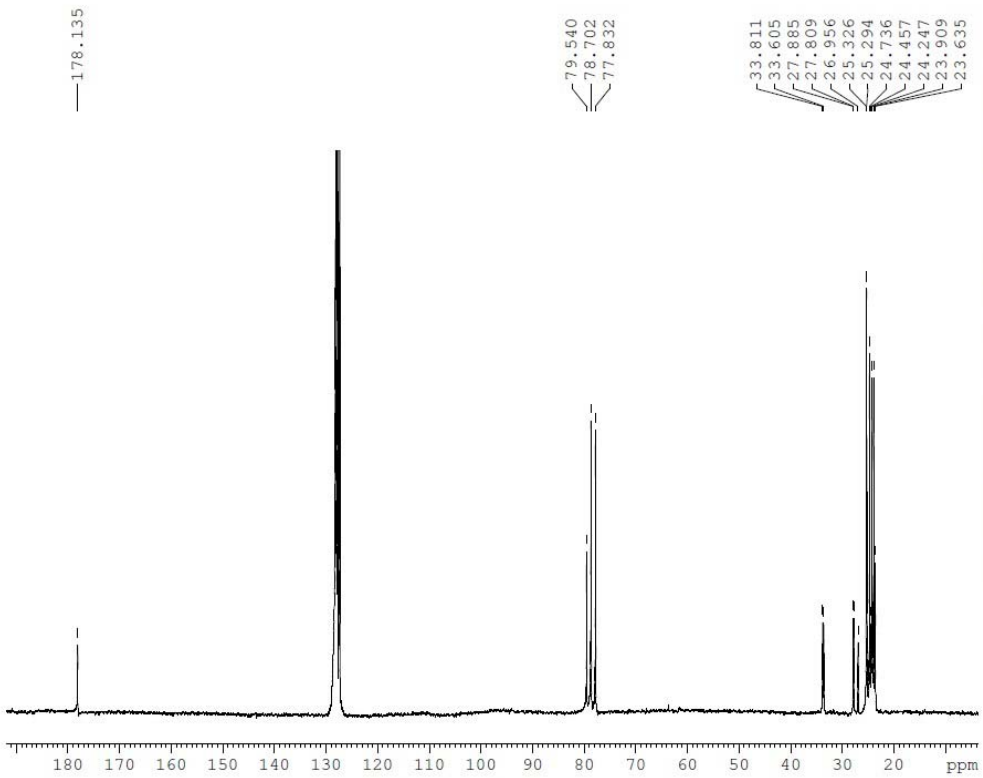

^{1}H NMR spectrum of **9**

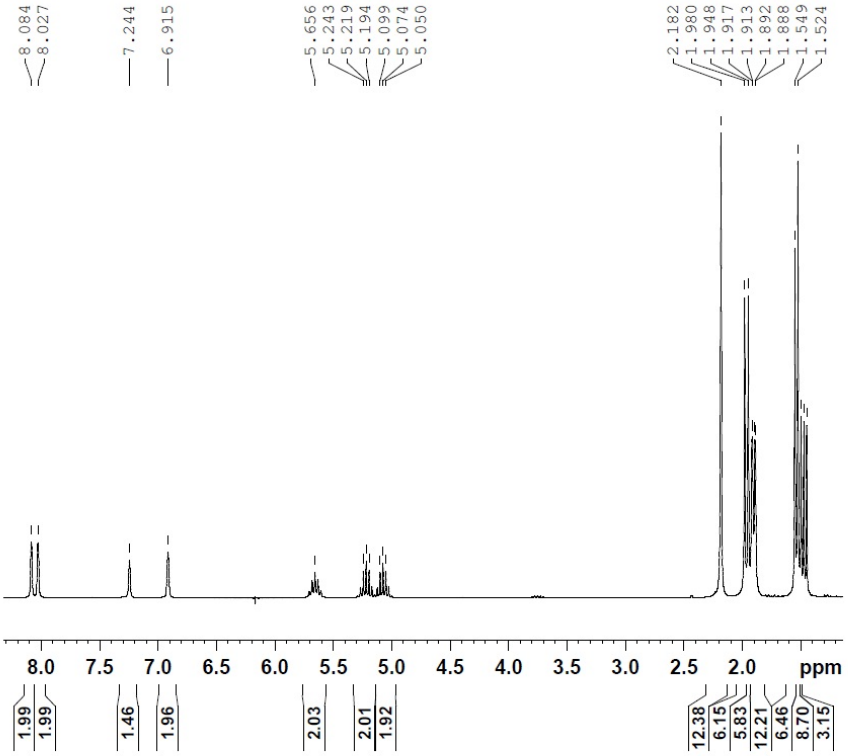

^{13}C NMR spectrum of **9**

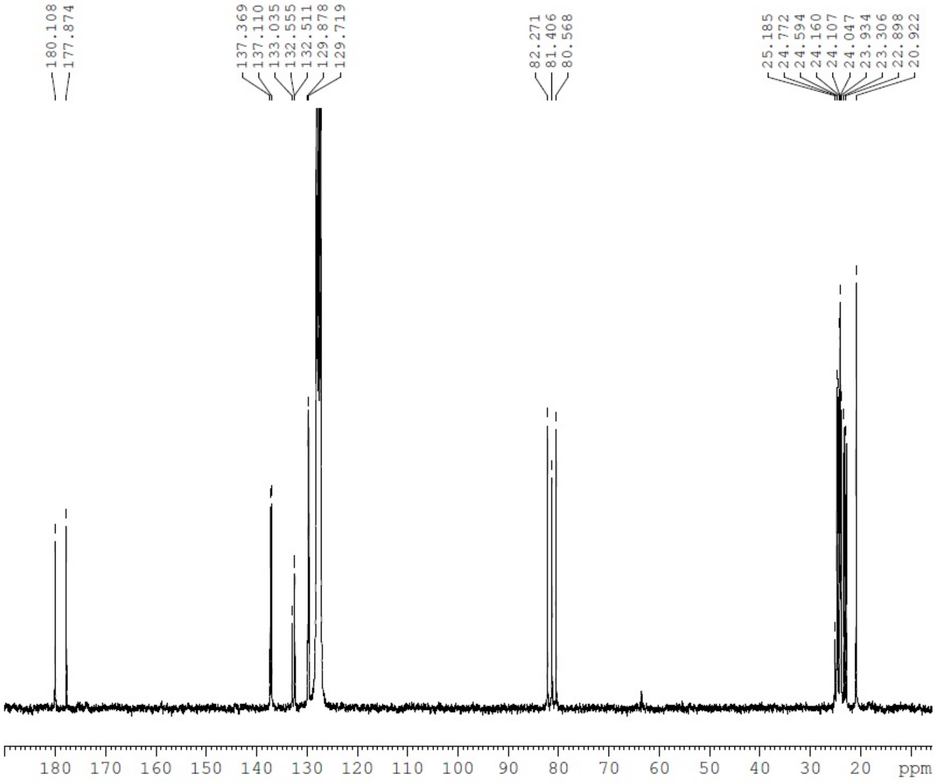

Supplement: Supplementary file 1 — miscellaneous_information [file ejic2014-2038-SD1.pdf]
